# Lactiplantibacillus plantarum 22 A-3 ameliorates leaky gut in mice through its anti-inflammatory effects

**DOI:** 10.1038/s41598-025-87428-3

**Published:** 2025-01-25

**Authors:** Takashi Kobayashi, Takaomi Kessoku, Michihiro Iwaki, Asako Nogami, Masato Yoneda, Satoru Saito, Yoshie Yamana, Yosuke Nishitani, Hiroshige Kuwahara, Atsushi Nakajima

**Affiliations:** 1 Department of Gastroenterology and Hepatology, Yokohama City University Graduate School of Medicine, Yokohama, Japan; 2 Department of Palliative Medicine, International University of Health and Welfare Narita Hospital, Chiba, Japan; 3 Department of Gastroenterology, International University of Health and Welfare Graduate School of Medicine, Chiba, Japan; 4 Department of Gastroenterology, Sanno Hospital, Tokyo, Japan; 5 Research Center, Maruzen Pharmaceuticals Co., Ltd., Hiroshima, Japan

**Keywords:** *Lactiplantibacillus plantarum 22A-3*, Probiotics, Lactic acid bacteria, Gut microbiota, Small intestine, Gastrointestinal diseases, Gastrointestinal system, Bacteria, Clinical microbiology

## Abstract

There are limited studies on the improvement of leaky gut with minor inflammation associated with various diseases. To explore the therapeutic potential of *Lactiplantibacillus plantarum 22 A-3*, a member of the *Lactobacillus* species, in addressing a leaky gut. *Lactiplantibacillus plantarum 22 A-3* was administered to a leaky gut mice model with low dextran sulfate sodium concentrations. The *Lactiplantibacillus plantarum 22 A-3*-treated group exhibited amelioration of increased intestinal permeability, as indicated by lower blood fluorescein isothiocyanate-dextran levels compared with that of the control group. Furthermore, the messenger RNA expression of interleukin-10, an anti-inflammatory cytokine, was upregulated in the small intestine of *Lactiplantibacillus plantarum 22 A-3*-treated mice. Moreover, forkhead box P3 was upregulated in the small intestine and colon following *Lactiplantibacillus plantarum 22 A-3* administration. Flow cytometry showed that forkhead box P3-positive regulatory T cells tended to increase in the small intestine and colon; however, this was not significant. Messenger RNA levels for the pro-inflammatory cytokines, interleukin-1 beta, and tumor necrosis factor-alpha showed no significant changes in the small intestine; however, their expressions significantly decreased in the colon. Blood fluorescein isothiocyanate-dextran levels showed that intestinal permeability also decreased in *Lactiplantibacillus plantarum 22 A-3*-dead bacteria. The bacterial component of *Lactiplantibacillus plantarum 22 A-3* ameliorates increased intestinal permeability through its anti-inflammatory effect in the intestinal tract and may be a novel treatment for leaky gut.

**Supplementary Information:**

The online version contains supplementary material available at 10.1038/s41598-025-87428-3.

## Introduction

Leaky gut syndrome is a condition in which intestinal permeability is increased, leading to intestinal tract leakage of pathogen-associated molecular patterns derived from intestinal bacteria, causing various symptoms and diseases^1^. These diseases include metabolic dysfunction-associated steatotic liver disease (MASLD), dementia, autoimmune conditions, allergies, and depression^2–6^. Specific intestinal bacteria can injure or protect intestinal endothelial cells, influencing overall intestinal permeability.

The intestinal bacterium *Lactiplantibacillus plantarum 22 A-3* (LP22A3) is a plant-derived lactic acid bacteria, discovered in pickles of the eggplant family^7,8^. Lactic acid bacteria are useful for improving the diversity of intestinal bacteria and intestinal environment. LP22A3, a prominent lactic acid bacteria strain, has demonstrated effectiveness in a mouse model of colitis, particularly that with gross colitis. Its potential application in treating inflammatory bowel disease is currently being explored^9^. However, studies on the amelioration of minor inflammation-induced leaky gut associated with various diseases are still limited. For example, administration of Lactobacillus and Bifidobacterium strains to obese mice, which have been linked to leaky gut, has been reported to improve serum cholesterol levels and steatotic liver disease^10^. However, it is not clarified whether intestinal permeability is involved in the improvement of these pathogenesis. There is a need to develop safe and effective treatments for leaky gut induced by minor inflammation, which is the cause of various diseases, and to elucidate the mechanisms of the disease.

The study aimed to determine the effectiveness of intestinal bacterium LP22A3 in improving leaky gut in mice.

## Methods

### Ethics approval

The experiment was approved by the Yokohama City University Animal Experiment Committee under approval number F-A-19-022. It was conducted following the ethical guidelines and regulations set by the Yokohama City University School of Medicine Ethics Committee.

### Bacteria

LP22A3 was cultured in de Mann, Rogosa and Sharpe broth and incubated overnight at 30 °C in an anaerobic chamber. Following incubation, bacterial cells were collected through centrifugation (4 °C, 990 × *g*, 10 min), resuspended in phosphate-buffered saline, and washed twice to remove the culture medium. The bacterial cells were lyophilized and made into viable bacteria (> 10¹⁰ cfu/g). For experiments involving dead bacteria, viable bacteria were inactivated by dry heat treatment at 90 °C overnight. The absence of viable bacteria was confirmed by the absence of colony formation on MRS agar plates. Lyophilized viable and dead bacteria were stored at -20 °C until use.

### Mouse model

In total, 72 male C57BL/6 mice were used in this study. Of these, 24 mice were used to develop the leaky gut model, 24 mice were used to evaluate the effects of LP22A3 administration, and 24 mice were used in experiments with heat-killed bacteria. Each experimental group consisted of 6 mice. Seven-week-old male C57BL/6 mice were obtained from CLEA Japan, Inc. (Tokyo, Japan). Upon arrival, the mice were housed in the animal facilities at Yokohama City University. They were provided with unrestricted access to standard laboratory food. Mice were anesthetized with an intraperitoneal injection of Somnopentyl (40 mg/kg) combined with Selactar (20 mg/kg) to ensure both anesthesia and analgesia. The animals were monitored closely for signs of distress or inadequate anesthesia. Mice were euthanized by gradual-fill CO2 inhalation, as per institutional guidelines, to minimize distress. Cervical dislocation was performed post-CO2 exposure to confirm euthanasia. To ensure the ethical treatment of animals and minimize unnecessary suffering, predefined humane endpoints were established for this study. Animals were monitored daily, and the following criteria were used to determine the need for euthanasia or other interventions: a persistent weight loss of more than 20% compared to baseline body weight; signs of severe dehydration; difficulty accessing food or water; visible bleeding; signs of paralysis, severe ataxia, or self-mutilation. Animals meeting any of these criteria were immediately euthanized using carbon dioxide (CO2) inhalation. Experiments were conducted following the ARRIVE guidelines to ensure transparency, reproducibility, and ethical treatment of animals. These procedures were approved by the Yokohama City University Animal Experiment Committee under permit number F-A-19-022.

### Development of the leaky gut model mice

Eight-week-old male C57BL/6 mice were randomly assigned to one of the four experimental groups (*n* = 6 per group). Each group received specific treatments for 7 days. The mice were subsequently sacrificed when they were 9 weeks old. The treatments administered to the different groups were as follows: normal drinking water, 0.05% dextran sulfate sodium (DSS), 0.5% DSS, or 3.0% DSS. Before the random allocation, all mice underwent a 1-week acclimatization period in the laboratory environment to ensure they were accustomed to their surroundings. On day 7 of treatment, after administering analgesia and sedation, blood samples were collected. Subsequently, the mice were euthanized, and their colons were carefully removed. The length of each colon was measured and recorded, and the number of ulcers present was counted to assess the degree of colon damage. The percentage of mice that developed colon ulcers observable under an optical microscope was then calculated.

Additionally, intestinal permeability was evaluated by measuring the blood concentration of fluorescein isothiocyanate (FITC)-dextran. This measurement was used to determine how easily substances could pass through the intestinal barrier. The group that was used to model a leaky gut exhibited increased intestinal permeability, as evidenced by higher blood levels of FITC-dextran, despite not showing obvious signs of colitis, such as ulceration or shortening of the colon. This indicated that while the intestinal barrier was compromised, resulting in increased permeability, there were no significant macroscopic changes to the colon structure typically associated with colitis.

### LP22A3 administration to the leaky gut mouse model

C57BL/6 mice (8 weeks old, male) were used in this study and were divided into four experimental groups (*n* = 6 per group). The experimental groups were treated with 0.5% DSS or normal drinking water for 7 days. This DSS concentration was determined based on results from the above study. Additionally, the mice were orally administered 1 × 10^9^ CFUs of LP22A3 or phosphate-buffered saline as vehicle control for 7 days. In the experiment with dead bacteria, mice were divided into four groups (*n* = 6 per group). They were administered the same bacterial dose with the same duration as that in the live bacteria experiment. Following the administration of analgesia and sedation on day 7 of treatment, blood samples were taken. The mice were then humanely euthanized, and their small intestines and colons were carefully dissected.

### Blood FITC-dextran levels

Four hours before sacrifice, each mouse was administered 600 mg/kg of FITC-dextran, obtained from Sigma-Aldrich (St. Louis, MO, USA). The FITC-dextran used had an average molecular weight of 4 kDa. This compound was given orally to the mice to allow for the assessment of intestinal permeability. After 4 h, the mice were anesthetized during sample collection. Blood samples were then obtained from the portal vein of each mouse. These blood samples were subsequently centrifuged at 4 °C and 800 × *g* for 15 min. The centrifugation process was performed to separate the plasma from the blood cells, and the resulting plasma was collected for further analysis. The quantification of intestinal permeability was performed by measuring the levels of FITC-dextran present in the plasma samples. This was accomplished using a fluorescence plate reader sourced from Promega (Madison, WI, USA) or TECAN (Männedorf, Switzerland). The fluorescence measurements were conducted with the plate reader set to excitation and emission wavelengths of 485 nm and 535 nm, respectively. These specific wavelengths were selected to optimize the detection of FITC-dextran fluorescence.

### Quantitative reverse transcription-polymerase chain reaction

RNA extraction from the colon and small intestine was performed using the RNeasy Midi Kit from Qiagen (Germantown, MD, USA). Following RNA extraction, reverse transcription to complementary DNA was executed using the SuperScript^®^ VILO™ MasterMix or the High Capacity RNA-to-cDNA Kit, both from Thermo Fisher Scientific (Waltham, MA, USA), following the manufacturer’s instructions. Messenger RNA expression levels were quantified using the Taqman method, using the FastStart Universal Probe Master with ROX from Roche Applied Sciences (Basel, Switzerland) or TaqMan^®^ Fast Advanced Master Mix from Thermo Fisher Scientific. Real-time polymerase chain reaction (PCR) was conducted using the StepOnePlus Real-Time PCR System or QuantStudio^®^ 3 RealTime PCR System, both from Thermo Fisher Scientific. Supplementaly Table 1 shows specific primer sequences used in this study. Primers for the genes interleukin (*Il)-10*, transforming growth factor β1 *(Tgf-β1)*, forkhead box P3 *(Foxp3)*,* Il-1β*, tumor necrosis factor-alpha *(Tnf-α)*, *Il-6*, Tight Junction Protein 1 *(Tjp1)*, *Tjp2*, Occludin *(Ocln)*, Claudin-2 *(Cldn2)*, *Cldn3*,* and Cldn4* were obtained from Sigma-Aldrich. Primers for the housekeeping gene, Beta-actin (Actb), were sourced from Thermo Fisher Scientific. The housekeeping gene *Actb* was used to normalize *Il-10*,* Tgf-β1*,* Foxp3*,* Il-1β*,* Tnf-α*, *Il-6*,* Tjp1*,* Tjp2*,* Ocln*,* Cldn2*, *Cldn3*,* and Cldn4* expression levels, ensuring the accuracy and reliability of the quantification process.

### Isolation of lamina propria lymphocytes

Lamina propria lymphocytes (LPLs) were isolated following a previously published protocol with several modifications to optimize the procedure^11,12^. The small intestine or colon was carefully excised from the mouse and then longitudinally opened to expose the inner surface. Furthermore, the tissue was washed with a solution containing 30 mM ethylenediaminetetraacetic acid while on ice, a step designed to remove epithelial cells. Following this washing step, the intestinal epithelium was carefully discarded, ensuring that only the lamina propria remained for subsequent processing. The remaining tissue was then treated with Roswell Park Memorial Institute 1640 medium from Nacalai Tesque (Nagoya, Japan), supplemented with 50 mg/mL collagenase D and 1 mg/mL DNAase, both of which were sourced from Roche. The tissue was incubated at 37 °C for 16 min to ensure adequate cell release without excessive degradation. After the incubation, the resulting cell suspension, which contained the released LPLs, was collected and filtered through a 70 μm cell strainer. The filtered cell suspension was then resuspended in 40% Percoll solution. This suspension was carefully layered on an 80% Percoll solution. The preparation was then centrifuged at 700 × *g* for 12 min.

### Flow cytometry

Isolated LPLs were used to analyze the presence and characteristics of CD25^+^ Foxp3^+^ cells in the small intestine and colon. The cells underwent a detailed staining process using fluorescently labeled antibodies to identify specific cell surface markers and intracellular proteins. The antibodies used for this staining included anti-CD3 (clone 17A2, BioLegend, San Diego, CA, USA), anti-CD4 (clone GK1.5, Thermo Fisher Scientific), anti-CD25 (clone PC61.5, Thermo Fisher Scientific), and anti-FOXP3 (clone FJK-16s, Thermo Fisher Scientific). Furthermore, the Foxp3/Transcription Factor Fixation/Permeabilization Concentrate and Diluent from Thermo Fisher Scientific were used to accurately detect intracellular Foxp3. Following the staining procedure, the outcomes were thoroughly analyzed using the Attune NxT flow cytometer (Thermo Fisher Scientific).

### Statistical analyses

Statistical analyses were conducted using the JMP Pro 15 software package developed by the SAS Institute (Cary, NC, USA). Notably, all data are expressed as the means ± standard error. Furthermore, the Student’s or Welch’s t-tests were used to compare normally distributed data between two groups. However, the Wilcoxon rank-sum test was employed for datasets that were not normally distributed. Furthermore, the Wilcoxon rank-sum test was used in situations where the comparison involved two related samples with non-normally distributed data. The Tukey–Kramer, Dunnett, and Steel–Dwass tests were used to compare data across three or more groups and were selected based on the data distribution. The permutational multivariate analysis of variance was also conducted to assess clustering shifts within the data. Statistical significance was set at *p* < 0.05.

## Results

### Establishment of leaky gut model mice with DSS

**Fig. 1:**
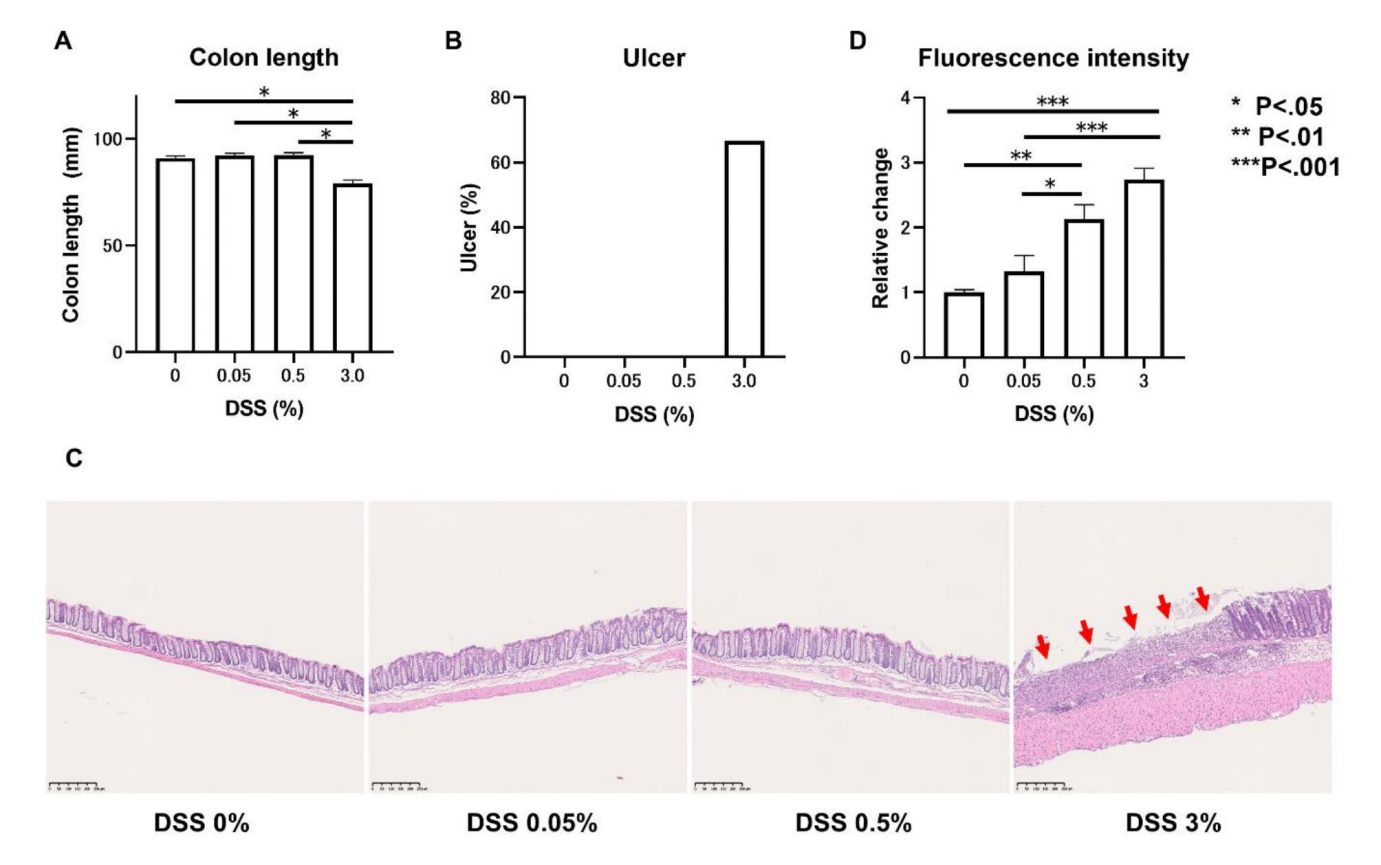
Assessment of DSS concentrations for the development of leaky-gut model mice. (**A**, **B**) Length of colon in mice administered varying concentrations of DSS. (**B**, **C**) Incidence of colon ulcers and hematoxylin-eosin staining of the colon. The ulcer is indicated with arrows. (**D**) FITC-dextran blood levels for the assessment of intestinal permeability. Data are the means ± SEM. *p < 0.05, **p < 0.01, ***p < 0.001.

Mice were administered normal drinking water, 0.05% DSS, 0.5% DSS, or 3.0% DSS for 7 days. The group receiving 3.0% DSS had significantly shorter colon length than those receiving 0.05% (*p* < 0.05) or 0.5% DSS (*p* < 0.05) (Fig. 1A). In addition, 60% of mice in the group receiving 3.0% DSS had colonic ulcers; however, none of the mice in the 0.05% or 0.5% groups had ulcers (Fig. 1B, C).

Blood FITC levels were significantly higher in the ≥ 0.5% DSS group than in the 0% or 0.05% DSS group (Fig. 1D), demonstrating that intestinal permeability increased after administering ≥ 0.5% DSS. Therefore, 0.5% DSS is the concentration that increases intestinal permeability without causing gross colitis, and a 0.5% DSS loading for 1 week was used to create the leaky gut model.

### Oral administration of LP22A3 improves gut permeability

**Fig. 2:**
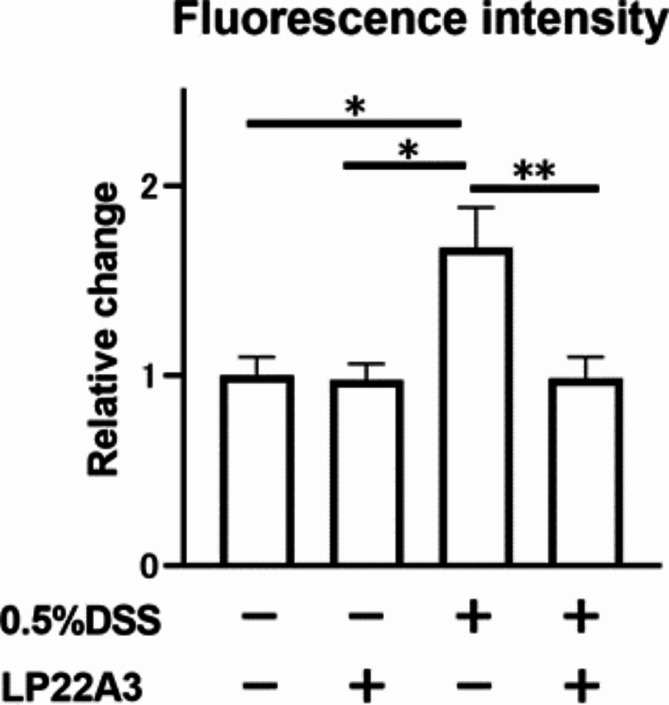
LP22A3 improves increased intestinal permeability in a mouse model of a leaky gut. *p < 0.05, **p < 0.01.

Furthermore, to investigate whether LP22A3 improves leaky gut, we examined blood FITC-dextran levels in leaky gut model mice with 0.5% DSS. There was no significant difference in blood FITC levels between the 0% DSS + Vehicle and 0% DSS + LP22A3 groups. However, the 0.5% DSS + Vehicle group had significantly higher blood FITC-dextran levels than the 0% DSS + Vehicle group (*p* < 0.05). In contrast, the 0.5% DSS + LP22A3 group had significantly lower blood FITC-dextran levels than the 0.5% DSS + Vehicle group (*p* < 0.01). The 0.5% DSS + LP22A3 group exhibited decreased blood FITC levels similar to that in the two 0% DSS groups (Fig. 2), suggesting that LP22A3 administration ameliorated increased intestinal permeability in the leaky gut model mice.

### Oral administration of LP22A3 induces anti-inflammatory cytokines in the gut

Furthermore, to examine the mechanism through which LP22A3 alters intestinal permeability, we evaluated the expression of anti-inflammatory cytokines and their associated transcription factors (IL-10, TGF-β, and Foxp3) and the expression of pro-inflammatory cytokines (IL-1β, TNF-α, and IL-6) in the small intestine and colon, respectively.

**Fig. 3:**
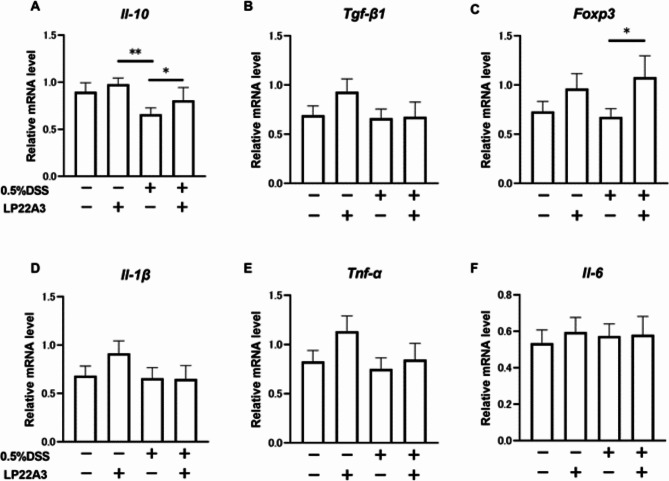
Effects of LP22A3 on the expression of anti-inflammatory and pro-inflammatory cytokines in the small intestine. (**A**–**C**) Effects of LP22A3 on anti-inflammatory cytokines *Il-10* and *Tgf-β1* and the master gene of regulatory T cells, *Foxp3*, in the small intestine. (**D**–**F**) Changes in the expression of the pro-inflammatory cytokines *Il-1β*, *Tnf-α*, and *Il-6* in the small intestine upon LP22A3 administration. *p < 0.05, **p < 0.01.

In the PCR analysis of small intestine LPLs, specifically targeting anti-inflammatory cytokines, IL-10 and Foxp3 expression was significantly increased in the 0.5% DSS + LP22A3 group compared with that in the 0.5% DSS + Vehicle group (*p* < 0.05), demonstrating that LP22A3 treatment induces these anti-inflammatory cytokines in the colon of leaky gut mice. However, there was no significant change in the expression of TGF-β (Fig. 3A–C). Alternatively, no significant difference was observed for pro-inflammatory cytokines in small intestinal LPLs in the 0.5% DSS + LP22A3 group compared with that in the 0.5% DSS + Vehicle group (Fig. 3D–F).

**Fig. 4:**
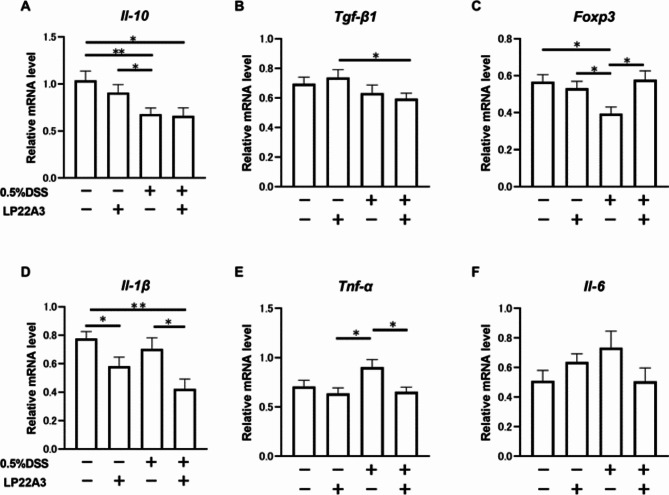
Effects of LP22A3 on the expression of anti-inflammatory and pro-inflammatory cytokines in the colon. (**A**–**C**) Effects of LP22A3 on anti-inflammatory cytokines *Il-10* and *Tgf-β1* and the master gene of regulatory T cells, *Foxp3*, in the colon. (**D**–**F**) Changes in the expression of the pro-inflammatory cytokines *Il-1β*, *Tnf-α*, and *Il-6* in the colon upon LP22A3 administration. *p < 0.05, **p < 0.01.

However, in the PCR analysis of colon LPLs, focusing on anti-inflammatory cytokines, there were no significant differences in the expression levels of IL-10 and TGF-β between the 0.5% DSS + LP22A3 and 0.5% DSS + Vehicle groups (Fig. 4A and B). Conversely, Foxp3 expression was significantly higher in the 0.5% DSS + LP22A3 group than in the 0.5% DSS + Vehicle group (*p* < 0.05), demonstrating that LP22A3 treatment induced Foxp3 in the colon of leaky gut mice. (Fig. 4C). Regarding pro-inflammatory cytokines, the expression of IL-1β and TNF-α was significantly higher in the 0.5% DSS + Vehicle group than in the 0% DSS + Vehicle group (*p* < 0.05), showing that 0.5% DSS administration significantly increased the expression of these pro-inflammatory cytokines. In addition, the expression of these two pro-inflammatory cytokines was significantly lower in the 0.5% DSS + LP22A3 group than in the 0.5% DSS + Vehicle group (*p* < 0.05), demonstrating that LP22A3 significantly suppressed the expression of these pro-inflammatory cytokines in the colon of leaky gut mice. (Figs. 4D and E). IL-6 expression was also downregulated in the 0.5% DSS + LP22A3 group compared with that in the 0.5% DSS + Vehicle group; however, this was not significant (Fig. 4F).

**Fig. 5:**
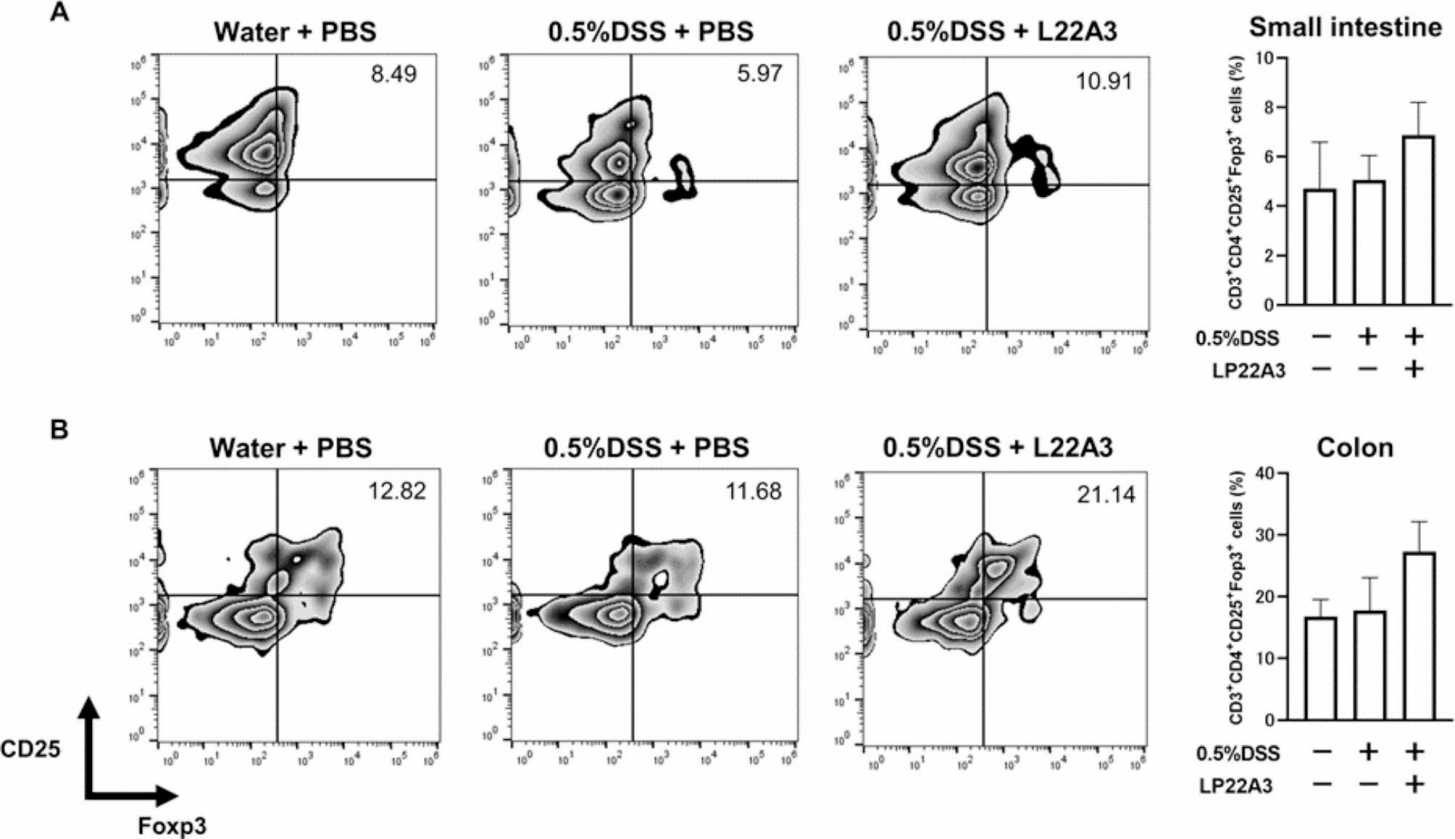
Analysis of regulatory T cell (Treg) counts in the intestinal mucosal lamina propria lymphocytes using flow cytometry. There was a trend towards increased Tregs upon LP22A3 administration in both the (**A**) small intestine and (**B**) colon, although this was not significant.

Foxp3^+^ regulatory T cells (Treg) counts were assessed using flow cytometry in LPLs of the small intestine and colon. In both organs, there was a non-significant trend toward higher cell counts in the 0.5% DSS + LP22A3 group than in the two groups without LP22A3 (Fig. 5). Therefore, in the small intestine and colon, the oral administration of LP22A3 showed a tendency to increase Foxp3^+^ Tregs in the LPL.

Furthermore, the gene expression of tight junction proteins (TJP1, TJP2, OCLN, CLDN2, CLDN3, and CLDN4) in the small intestine and colon was compared between the 0.5% DSS + LP22A3 group and the 0.5% DSS + Vehicle group. Although no significant differences were observed, there was a tendency for increased expression of four genes (TJP2, CLDN2, CLDN3, and CLDN4) in the small intestine and all six genes in the colon in the 0.5% DSS + LP22A3 group compared with the 0.5% DSS + Vehicle group (Supplementary Figs. S1, S2).

### Oral administration of dead LP22A3 ameliorates increased gut permeability

**Fig. 6:**
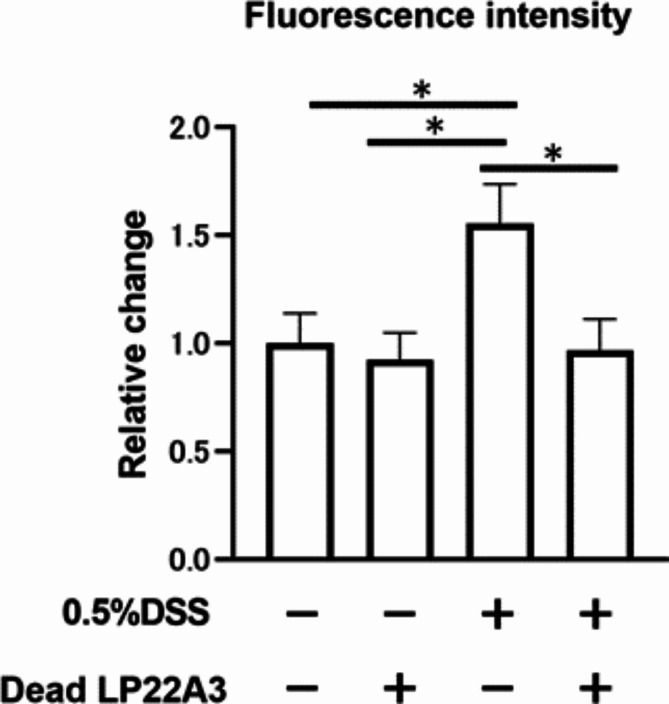
Improved intestinal permeability upon administration of dead LP22A3 in a leaky gut mouse model. *p < 0.05.

Furthermore, to determine whether the alterations in intestinal permeability induced by LP22A3 were due to bacterial components or metabolites, heat-inactivated LP22A3 cells were administered to mice. The 0.5% DSS + dead bacteria group showed significantly lower blood FITC levels than the 0.5% DSS + vehicle group (*p* < 0.05) (Fig. 6). Therefore, LP22A3 ameliorated the increased intestinal permeability induced by 0.5% DSS even when dead bacteria were administered. This suggests that the effect of LP22A3 in altering intestinal permeability may be due to its bacterial components rather than its metabolites.

## Discussion

In the present study, LP22A3 ameliorated the increased intestinal permeability in a leaky gut mice model induced by 0.5% DSS. Oral administration of LP22A3 also decreased the expression of pro-inflammatory cytokines such as IL-1β and TNF-α in the colon. In addition, the expression of IL-10, an anti-inflammatory cytokine, was increased in the small intestine, and the expression of Foxp3, a marker of Treg, was increased in both the small intestine and colon. Flow cytometry showed a trend toward increased Foxp3 + Treg in the small intestine and colon upon oral administration of LP22A3. LP22A3 has a therapeutic effect on leaky gut, which may be mediated by its anti-inflammatory properties. In addition, the intestinal permeability evaluated using FITC was improved with both live and dead bacteria, suggesting that bacterial components, not metabolites, improve a leaky gut.

DSS is an agent primarily used in mice models of colitis^13–15^. Typically, these mice models use DSS in higher concentrations or for longer periods than those used in the present study. In the early development of the DSS colitis model, Okayasu et al. administered 5% DSS for 7 days and repeated this for five additional cycles^16^. In recent reports, acute colitis is typically induced with 2–5% DSS administered continuously for 4–9 days^17^. Notably, in our model, a low concentration of DSS (0.5%) administered for 7 consecutive days caused leaky gut syndrome without gross colon changes, such as shortening or ulceration.

LP22A3 reportedly ameliorates colitis in mice with gross inflammation findings, such as a shortened colon; however, whether it ameliorates the leaky gut associated with minor inflammation, which is thought to be involved in various systemic diseases, remains unclear^9^. In the present study, we used a mice model of leaky gut syndrome, a condition frequently associated with minor inflammation. Thus, this model provides means to assess the effectiveness of treatments for leaky gut syndrome in the presence of minor intestinal inflammation.

The pro-inflammatory cytokines TNF-α and IL-1β cause a decreased expression and redistribution of tight junction proteins in the intestinal mucosa, resulting in increased intestinal permeability^18–20^. Pro-inflammatory cytokines also induce apoptosis of the intestinal mucosa and increase intestinal permeability^21^, thereby increasing intestinal permeability. Therefore, LP22A3 potentially improves the leaky gut by exerting its anti-inflammatory effects on the intestine.

Treatment with LP22A3 has shown to significantly increase the expression of Foxp3, a marker of Tregs, in the small intestine and colon^22^. Consequently, LP22A3 probably has anti-inflammatory effects on the small intestine and colon.

TGF-β is an anti-inflammatory cytokine that induces Treg differentiation^21^. *Clostridium butyricum*, an intestinal bacterium, promotes TGF-β secretion through dendritic cells and Treg differentiation in the intestinal mucosa^23–25^. However, LP22A3 did not show increased TGF-β expression in the small intestine and colon. Notably, various other factors, including IL-6, IL-23, IL-33, and retinoic acid derivatives, can induce Tregs^26,27^. Therefore, LP22A3 may be involved in Treg differentiation in a manner distinct from *Clostridium butyricum*.

Foxp3 was significantly upregulated in the small intestine and colon, whereas IL-10, an anti-inflammatory cytokine whose expression is upregulated by Foxp3, was significantly elevated only in the small intestine^28^. The anti-inflammatory effects of LP22A3 are primarily initiated in the small intestine and subsequently extend into the colon^9^. Therefore, variations in the locations of increased IL-10 expression may indicate differences in the sites where LP22A3 initiates its action.

In addition, differences were observed between the small intestine and colon concerning the inhibitory effect of LP22A3 on the expression of pro-inflammatory cytokines. This disparity may be attributed to the choice of the mouse model, as DSS is primarily used in colitis models. DSS is mainly toxic to colon epithelial cells and causes increased expression of pro-inflammatory cytokines, such as IL-1β, TNF-α, and IL-6 in the colon epithelium^29^. The leaky gut model used in this study also showed significantly increased expression of pro-inflammatory cytokines such as IL-1β and TNF-α and a trend toward increased expression of IL-6 compared with mice receiving normal water. However, in the small intestine, the administration of low concentrations of DSS did not significantly increase the expression of these pro-inflammatory cytokines. In this leaky gut model using DSS, the expression of pro-inflammatory cytokines was increased solely in the colon, with no corresponding increase in the small intestine. Consequently, the observed suppression of pro-inflammatory cytokines may be specific to the colon.

Intestinal bacteria and probiotics reduce intestinal inflammation and improve intestinal permeability^9,30,31^. However, existing studies have employed more severe models of colon inflammation, with limited reports on treatment for mild leaky gut characterized by the absence of subjective symptoms. LP22A3 administration did not significantly increase the expression of pro-inflammatory cytokines in either the small intestine or colon compared with mice receiving the vehicle in this study. This suggests that the bacteria can be safely administered.

This study has some limitations. The molecular mechanism underlying the improvement in intestinal permeability has not been fully elucidated, with potential latent unknown factors. In particular, it remains unclear which specific components of LP22A3 contribute to the improvement of leaky gut. A more detailed comparison of the effects of live and heat-killed bacteria may provide stronger evidence for the mechanisms underlying the effects of LP22A3. Additionally, it is unclear whether LP22A3 exhibits similar effects in humans owing to differences in the immunological mechanisms and intestinal microbiota between humans and mice. Therefore, further studies are warranted to clarify these issues.

## Conclusions

LP22A3 demonstrated anti-inflammatory effects and improved intestinal permeability in a mouse model of leaky gut using low concentrations of DSS without apparent adverse effects. Therefore, LP22A3 is expected to be a novel therapeutic approach for leaky gut syndrome, which is associated with various diseases.

## Electronic supplementary material

Below is the link to the electronic supplementary material.

Supplementary Material 1

Supplementary Material 2

Supplementary Material 3

Supplementary Material 4

## Data Availability

The data that support the findings of this study are available from the corresponding author upon reasonable request.
