# Supplementary material for: Lactiplantibacillus plantarum 22 A-3 ameliorates leaky gut in mice through its anti-inflammatory effects
Source: Sci Rep. 2025 Jan 25;15:3264. doi: 10.1038/s41598-025-87428-3 (PMC11762275; doi:10.1038/s41598-025-87428-3)
Supplement: Supplementary file 1 — Supplementary Material 1 [file 41598_2025_87428_MOESM1_ESM.docx]

**Supplementary Table 1** Primers used in gene expression assays.

| **Assay Target** |  | |
| --- | --- | --- |
| *Il-10* | **Probe** | TCTTCACCTGCTCCACTGCCTTGCTCT |
|  | **Forward** | TGAGGCGCTGTCATCGATTTC |
|  | **Reverse** | CCTTGGTCTTGGAGCTTATTAAAATC |
| *Tgf-β* | **Probe** | TTCAGCCACTGCCGTACAACTCCAGT |
|  | **Forward** | CCCACTGATACGCCTGAGTG |
|  | **Reverse** | AAGCCCTGTATTCCGTCTCC |
| *Foxp3* | **Probe** | CTGTGCTCCAAGTGCGTCCACTGGA |
|  | **Forward** | GCATCAGCTCTCCACTGTGG |
|  | **Reverse** | AGCAGAAGGTGGTGGGAGG |
| *Il-1β* | **Probe** | AGCAGCCCTTCATCTTTTGGGGTCCG |
|  | **Forward** | TGGCAACTGTTCCTGAACTCAA |
|  | **Reverse** | ACAGCCCAGGTCAAAGGTTTG |
| *Tnf-α* | **Probe** | AGGGGCCACCACGCTCTTCTGTCTAC |
|  | **Forward** | GCCTATGTCTCAGCCTCTTCTC |
|  | **Reverse** | AGGCCATTTGGGAACTTCTCATC |
| *Il-6* | **Probe** | ACAATCAGAATTGCCATTGCACAACTCTTT |
|  | **Forward** | GTTCTCTGGGAAATCGTGGA |
|  | **Reverse** | TTCTGCAAGTGCATCATCGT |
| *Actb* | **Catalog number** | 4352341E |
