# Supplementary material for: Lactiplantibacillus plantarum 22 A-3 ameliorates leaky gut in mice through its anti-inflammatory effects
Source: Sci Rep. 2025 Jan 25;15:3264. doi: 10.1038/s41598-025-87428-3 (PMC11762275; doi:10.1038/s41598-025-87428-3)
Supplement: Supplementary file 2 — Supplementary Material 2 [file 41598_2025_87428_MOESM2_ESM.docx]

**Supplementary Fig. S1**

Gene expression of tight junction proteins in the small intestine of mice. (A) Tjp1, (B) Tjp2, (C) Ocln, (D) Cldn2, (E) Cldn3, (F) Cldn4. N.S indicates not significant.

**Supplementary Fig. S2**

Gene expression of tight junction proteins in the colon of mice. (A) Tjp1, (B) Tjp2, (C) Ocln, (D) Cldn2, (E) Cldn3, (F) Cldn4. N.S indicates not significant.
