# Supplementary figures and images for: Lactiplantibacillus plantarum 22 A-3 ameliorates leaky gut in mice through its anti-inflammatory effects
Source: Sci Rep. 2025 Jan 25;15:3264. doi: 10.1038/s41598-025-87428-3 (PMC11762275; doi:10.1038/s41598-025-87428-3)

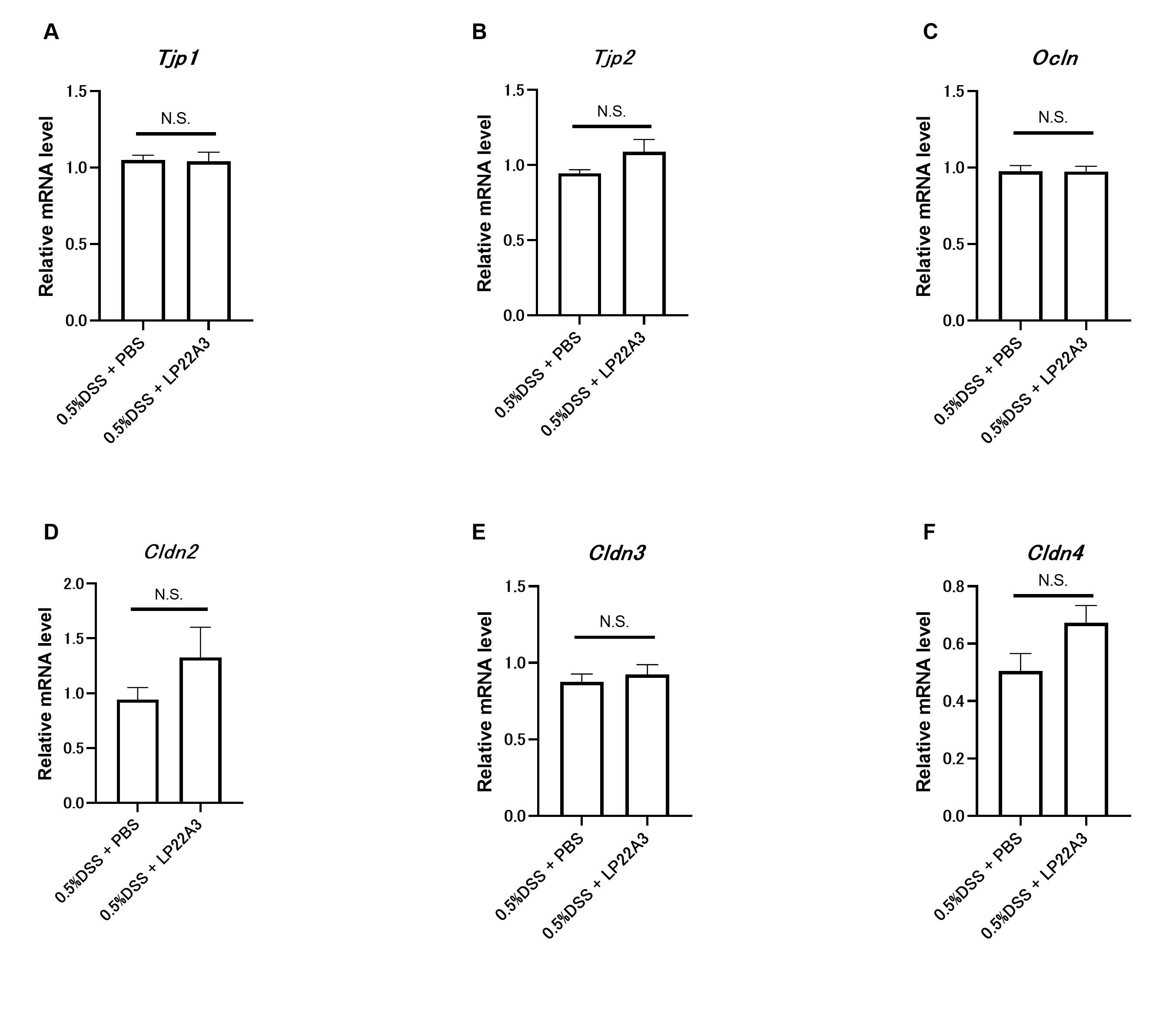

Supplement: Supplementary file 3 — Supplementary Material 3 [file 41598_2025_87428_MOESM3_ESM.tif]

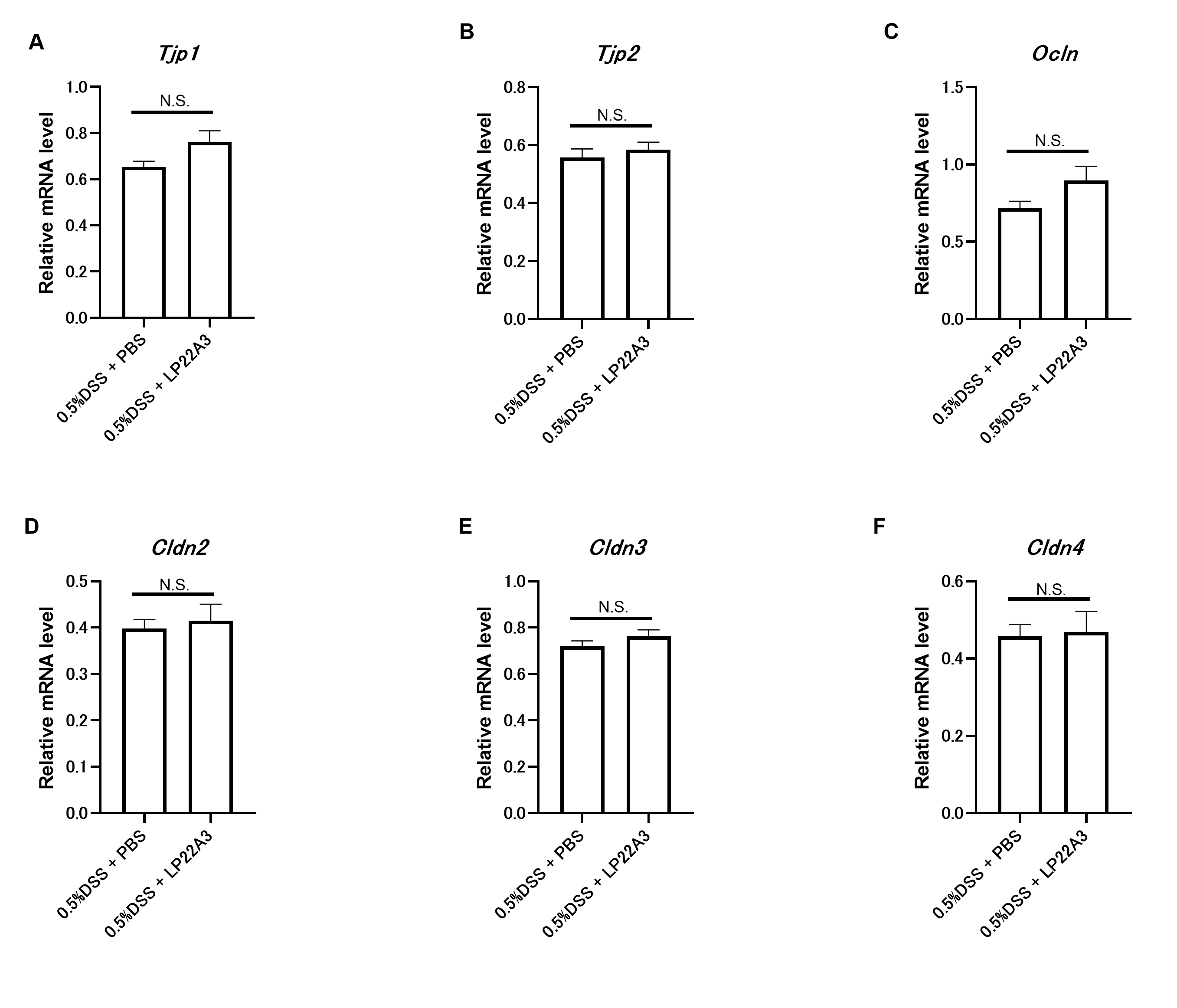

Supplement: Supplementary file 4 — Supplementary Material 4 [file 41598_2025_87428_MOESM4_ESM.tif]
